# Reducing microbial airborne contamination and particulate matter using different oral suctions in dental clinic: A randomized controlled clinical trial

**DOI:** 10.1016/j.sdentj.2023.11.029

**Published:** 2023-11-28

**Authors:** Abraar Bannan, Iman Kamal, Naief H. Al Makishah, Zuhair S. Natto

**Affiliations:** a Department of Community Medicine, Faculty of Medicine, King Abdulaziz University, Jeddah, Saudi Arabia; b Department of Public Health, Faculty of Medicine, AlAzhar University, Egypt; c Department of Environmental Science, Faculty of Environmental Sciences, King Abdulaziz University, Jeddah, Saudi Arabia; d Department of Dental Public Health, Faculty of Dentistry, King Abdulaziz University, Jeddah, Saudi Arabia

**Keywords:** Infection, Control, Microbial air quality, Suction, Intraoral, Extraoral

## Abstract

**Aim:**

This study aimed to assess oral suction devices in declining microbial airborne contamination level and particulate matter.

**Materials and methods:**

This open-label randomized clinical trial was conducted in an educational hospital with 50 participants above 18 years of age, who had scheduled an appointment at a dental hygienist clinic for scaling procedure. Particulate matter and microbial airborne contamination levels were taken at the beginning for 15 min and during of scaling procedure. Participants were randomized to five groups: low suction, high & low suction, intraoral suction (IOS), extra-oral suction (EOS) & low suction, and IOS & EOS. Repeated measured ANOVA analysis was carried out using STATA version 13.

**Results:**

Participants had aged 34.4 ± 8.1 years and the average simplified oral hygiene index was 3.5 ± 1.2. Microbial airborne contamination level for each intervention group was different to baseline; low suction, intraoral suction, high & low suction, EOS & low suction, and EOS and intraoral suction were 1089 ± 610, 296.3 ± 321.2, 43.8 ± 52.1, 17.3 ± 7.3 and 14.3 ± 3.9, respectively [P value < 0.05]. Particulate matter shows evidence of no significant difference among oral suctions [P value > 0.05].

**Conclusion:**

Low or intraoral suction was not enough to reduce microbial airborne contamination for better infection control, practitioners highly recommended to use combination of suction devices.

**Clinical relevance:**

Using extra-oral with intra-oral suction, or extra-oral suction with low section, or high & low suction are potentially better in microbial airborne contamination reduction compared with low or intraoral suction only.

**Trial registration:**

Clinicaltrials.gov (NCT05848245) on April 14, 2023.

## 1 Introduction

Aerosols are fluid or solid particles (Boucher, 2015) while bioaerosols are biological origin particles (Hirst, 2020). These particles which less than 50 µm diameter are usually suspended in the air for an extended period before resting on surfaces or entering the respiratory tract (da Silva, 2020). In the dental clinic, the dental team is exposed to infectious droplets through direct contact with the body fluid of the patient, and contact with environmental surfaces or instruments (Organization, 2020). Dental aerosol might be not easy to measure. However, many studies assess bacterial count using the cultures technique (Zemouri et al., 2017). Particles can be inhaled and held on the human lung’s and decrease lung function and lung capacity (Wyatt et al., 2020). Dental instruments and procedures generate various air-borne contamination amounts, the source of bioaerosol produce ultra-sonic scaler, air-driven high-speed handpiece, three way syringes, and air water syringes (Zemouri et al., 2017).

Using a personal protective barrier (PPE) would be prevented spatter droplets. However, using surgical face mask with or without eyes protection increase the potential risk of infection more than N95 respirator (Bischoff et al., 2011). There are new devices nowadays that might reduce contamination in the air such as intraoral suction [IOS] and extra-oral suction (EOS). Most of the study was concerned with the effectiveness of EOS, particularly during the pandemic COVID-19. Although rubber dam could be safer to use to limit the spread of contaminated water. However, it is not suitable to be used in all procedures and some patients (Matys and Grzech-Leśniak, 2020). There were some studies evaluating the influence of EOS, and they found EOS was effective in reducing droplets (Chavis et al., 2021, Ehtezazi et al., 2021, Graetz et al., 2021, Makhsous et al., 2021, Senpuku et al., 2021, Shahdad et al., 2021, Yang et al., 2021). A vitro study showed using IOS was effective by 88 % in reducing aerosol compared to other groups.(Matys and Grzech-Leśniak, 2020) The triangle area between dental staff and patient was recorded as the highest level of aerosol. Also, they found using high and low suction minimized the aerosol level, and EOS reduced it further down the baseline reading (Yang et al., 2021). However, most of these study (Chavis et al., 2021, Ehtezazi et al., 2021, Graetz et al., 2021, Shahdad et al., 2021, Yang et al., 2021) was performed in vitro but still it is need more investigations in vivo.

Lacking specific standards classification designed for air quality in dental clinic brings us to be more concerned about declining the exposure hazard to the greatest extent. Therefore, a comparison between interventions that may enhance the air quality could give a suggestion for a better environment. Infection control should be carried out to the maximum level to provide a safe environment in a dental clinic. Controlling aerosol and bioaerosol generated through a different procedure is important to patients and dental staff to reduce transmission of infectious disease. To the best of our knowledge, previous studies were interested in one outcome either microbial contamination or PM, some of the studies were done in humans or in vitro and included either a certain suction device or a small sample size. This study was more comprehensive as it included two outcomes and five different oral suctions in patients. This study aimed to assess the effectiveness of different dental suctions that could be contributed to decreasing the risk of particulate matter and microorganisms that arise from a patient mouth to indoor air in the dental clinic.

## 2 Materials and methods

**Fig. 1:**
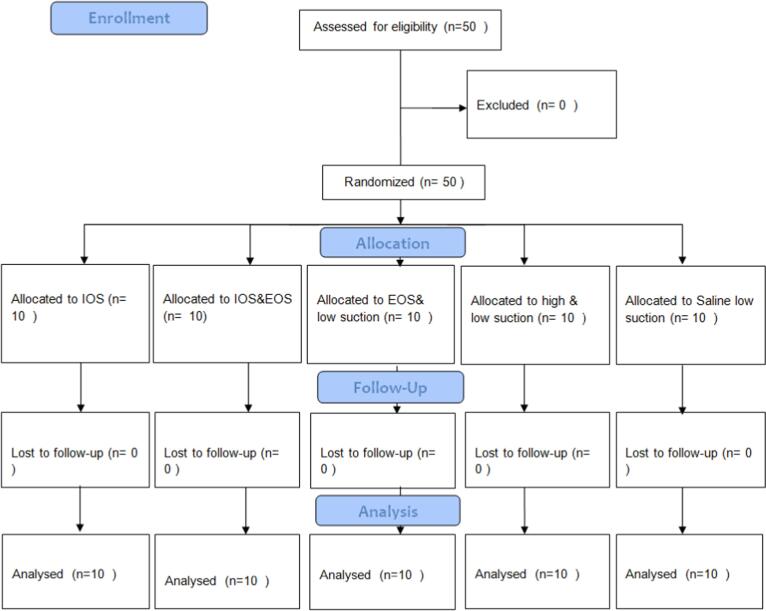
CONOSRT flow diagram (*IOS: Intraoral suction; EOS: extra-oral)*.

This is an open-label, randomized controlled clinical trial. It was approved by the ethical committee, faculty of dentistry at King Abdulaziz University (351–12-21) and followed Helsinki declaration. It was registered in clinicaltrails.gov (NCT05848245). Consent form was obtained presented by investigator for each subject before experimental trial. CONSORT was followed (Fig. 1). The study was started from May until December 2022.

### 2.1 Study population

50 Adult healthy participants above 18 years of age, who had scheduled an appointment at a dental hygienist clinic for scaling procedure were included. Each participant had to have at least one score of 2 or 3 in one sextant according to the partial community periodontal index of treatment needs (PCPITN) (Tanik and Gül, 2020). 20 teeth or less, presence of soft or hard tissue lesions, pregnant women, orthodontic patients, and partial denture wearers were excluded. Non-probability convenience sample was equally allocated randomly. Concealment was achieved by using sealed opaque numbers envelope in a container. Each participant select one number before entering to dental clinic using simple randomization, and it is one session for each patient.

### 2.2 Study area and setting

This study was conducted in enclosed room width = 247 cm, length = 362 cm, height = 283 cm, at King Abdulaziz dental hospital, Jeddah, Saudi Arabia. Humidity ranged between 46 % and 59 % and temperature ranged from 20.6 °C to 25.3 °C. Water in the clinic had been checked to be free of microorganisms using a Sartorius membrane filter device at the beginning of the study. Measuring particulate matter numbers was recorded using a digital dust monitor (model 3443, KANOMAX.USA INC., made in Japan) and air microbiology sample was collected using active method sampling (Sampl’air Lite, ASE A BioMérieux SA).

### 2.3 Intervention

Five interventions groups were included; A: IOS, B: IOS&EOS, C: EOS& low suction, D: high & low suction and E: low suction only (Fig. 1). All intervention devices were used as conventional way; low suction was hanged on the patient's mouth and moved thoroughly as needed, high suction was moved with scaler movement, and EOS where on the right-hand side of patient, power was turned on to level 10, and the suction cone was facing patient's mouth and it was away from its around 10–15 cm.

### *2*.4 Data collection and clinical examination

The first use of the room was for our trials after left it all night without work. Routine dental clinic disinfection was performed. Baseline samples were taken for 15 min before the patient and dental hygienist came inside the room while observer was present. Scaling procedure using a sonic scaler for the full mouth was completed within 15 min and pre-procedure rinse was not instructed for participants. The door was kept closed during sampling time. All dental procedures were carried out by one dental hygienist who had 5 years of experience, during the procedure was set in a 12o'clock position and wore PPE. Moreover, no one was blinded. The follow up samples were collected around 20 min after collecting baseline samples.

**Fig. 2:**
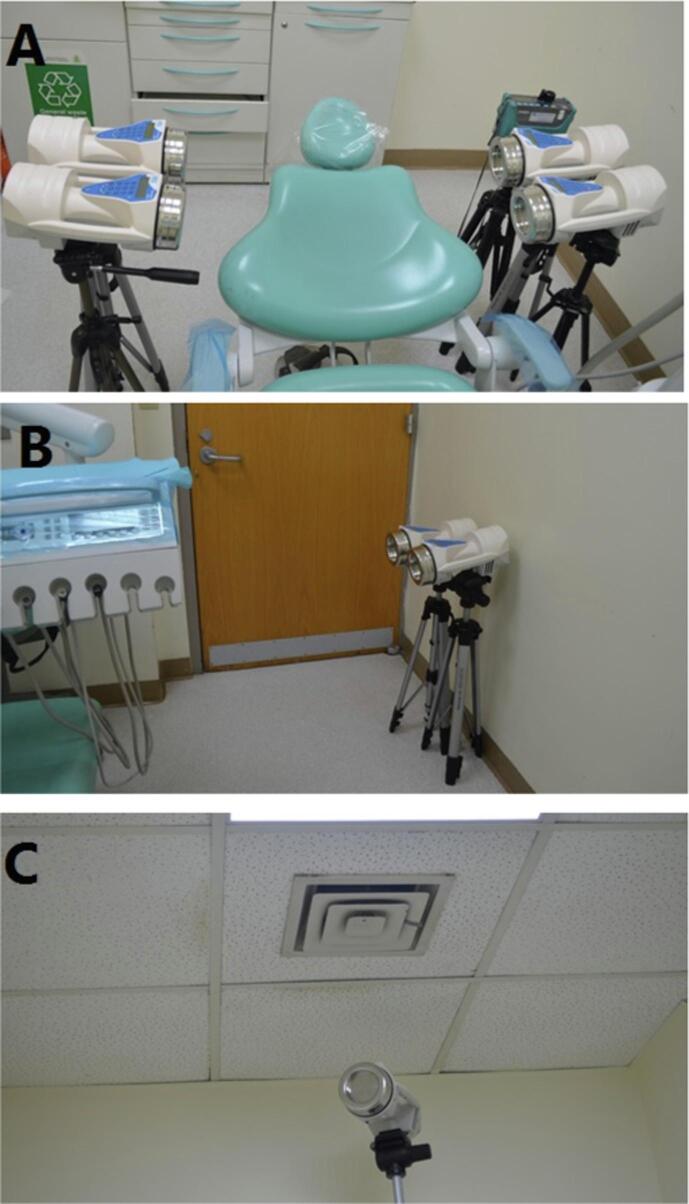
Distribution of microbial air sample devices and digital dust monitor around the dental chair A: at the nearest level to the patient, B: at the furthest point in the room which was near the door, C: near air conditioning.

Six microbial sampler devices were distributed at several locations in room above a floor distance of 75 cm; four devices were around the patient and two devices were at the farthest point from patient (Fig. 2A, 2B). Another two devices were placed near the air conditioning (AC) 260 cm above floor level as a control sample to AC microbial agents at the beginning of baseline sampling (Fig. 2C). Digital dust monitor was positioned at 1o'clock position 85 cm from the floor (Fig. 2A). The baselines samples were taking from closed dental room in the morning for 50 days before start any dental procedure.

For clinical examination, simplified oral hygiene index (OHI-S), simplified debris index (DI-S) and simplified calculus index (CI-S)(Bowen et al., 2020) were measured for each participant by dental hygienist. Dental hygienist was performed calibration test for dental indices. Test and retest for intra-reliability was one week apart - one in the morning and one in the afternoon, it was calculated using Pearson correlation coefficient for community periodontal index of treatment needs (r = 0.75) and simplified calculus index (r = 0.94).

### 2.5 Preparation and incubation

Nutrient agar media (BioLife Solutions inc.) was prepared based on the manufacturer's instructions. Duplicated samples were incubated for 48 h at a temperature of 37 °C. All the steps was conducted by one trained graduate dentist (AB) to use all equipment’s that included in the study. Moreover, she received training in preparation media and counting colonies from a microbiologist.

### 2.6 Sample size calculation and statistical analysis

Data entry and intent to treat statistical analysis were carried out using STATA version 13. Sample size power was calculated; the least power was 0.75 in the first group comparison and the greatest one was 0.99 in the last group comparison based on a preliminary data was conducted on 3 patients. All continuous variables were expressed as mean and standard deviation. All categorical variables were described as frequency and percentage. Repeated measured ANOVA was used to compare the difference in mean PM and microbial airborne contamination between the baseline reading and test. Comparing the mean of microbial airborne contamination, age, DI-S, CI-S, OHI-S, and intervention groups was performed using one-way ANOVA. Moreover, fisher's exact test for intervention groups and gender. All the used analyses were met the statistical assumptions. 95 % Confidence Interval and 0.05*P* value were considered significant levels.

## 3 Results

### 3.1 Participant's characteristic

A total of 50 participants were recruited, the average age was 34.4 ± 8.1 ranging from 18 to 52 years. 58 % of participants were males and 42 % were females. PCPITN revealed higher reading of score 2 or 3 in all sextants [score 2: 42–80 %, score 3: 14–46 %] except sextant 2 the highest reading scores were 1 [20 %] or 2 [66 %]. Total score for OHI-S was 3.5 ± 1.2, DI-S was 1.9 ± 0.6, and CI-S was 1.6 ± 0.6. There were no statistical significant between groups at baseline regarding DI-S, CI-S and OHI-S (Table 1).

**Table 1:** DI-S, CI-S and OHI-S distribution of participants among intervention groups at baseline.

Group	DI-S		CI-S		OHI-S	
	Mean ± SD	*P* value	Mean ± SD	P value	Mean ± SD	P value
Intraoral suction	2.0 ± 0.6	0.83	1.8 ± 0.7		3.8 ± 1.4	
EOS and intraoral suction	1.7 ± 0.6		1.5 ± 0.7		3.3 ± 1.4	
EOS and low suction	1.8 ± 0.6		1.5 ± 0.6	0.76	3.3 ± 1.4	0.76
High and low suction	1.9 ± 0.6		1.6 ± 0.5		3.5 ± 0.9	
Low suction	2.0 ± 0.6		1.8 ± 0.6		3.8 ± 1.2	

SD: standard deviation; EOS: extra-oral; DI-S: Simplified calculus index; DI-S: Simplified debris index; OHI-S: simplified oral hygiene index.

Statistical analysis carried out using one-way ANOVA test.* P value < 0.05.

### 3.2 Microbial airborne contamination level

**Fig. 3:**
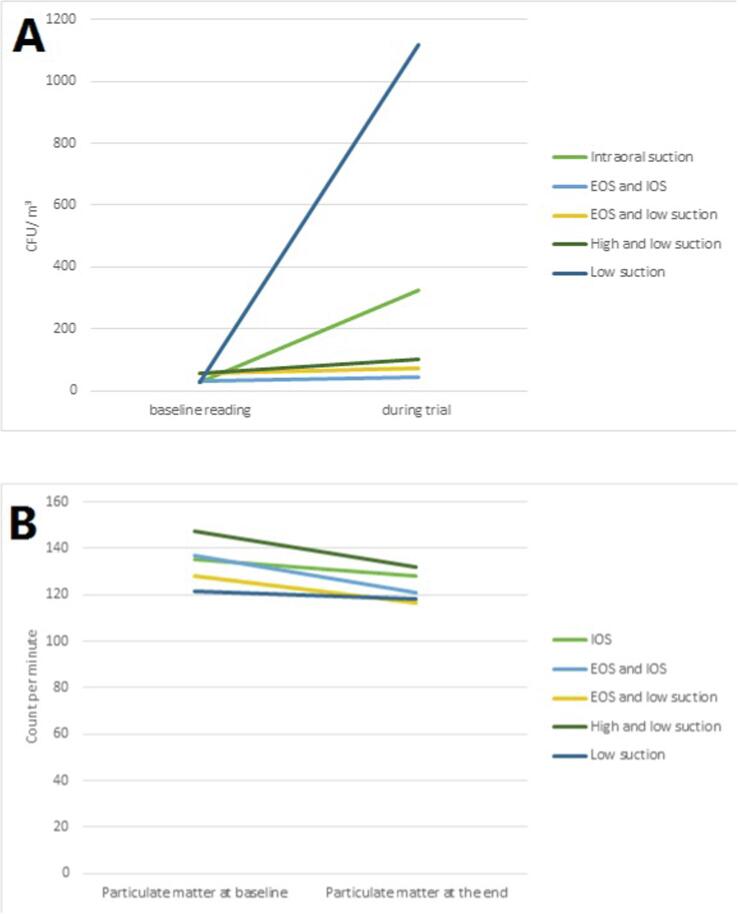
Comparison between A: microbial airborne contamination level (CFU/m^3^), B: particulate matter (count per minutes), at baseline reading and after trial among different oral suctions group (*IOS: Intraoral suction; EOS: extra-oral)*.

The average microbial airborne contamination level in the morning before any procedure started for 50 days was 40.3 ± 27.4. There was evidence of a significant difference in microbial airborne contamination level between the baseline sample and trial sample for intervention groups, *P* value < 0.001 (Fig. 3A).

Using the delta method, the low suction group and the IOS group were statistically significant [*P* value < 0.001] while other groups were not statistically significant [*P* value > 0.05]. The mean difference in the baseline sample and trial sample for the low suction group and the IOS group were 1089 ± 610 and 296.3 ± 321.2, respectively (Table 2). The different means among the baseline sample and trial sample; for the high and low suction group were 43.8 ± 52.1 while EOS and low suction were 17.3 ± 7.3 and EOS& IOS were 14.3 ± 3.9 (Table 2 and Fig. 3A).

**Table 2:** Comparison of microbial airborne contamination level (CFU/m^3^) and particulate matter between intervention groups.

Intervention groups	Baseline	During treatment	MD ± SD	*P* value
**Microbial airborne contamination level (CFU/m^3^)**				
Intraoral suction group	28.7 ± 15.0	325.1 ± 336.2	296.3 ± 321.2	0.005*
EOS and intraoral suction group	31.2 ± 10.8	45.6 ± 14.7	14.3 ± 3.9	0.887
EOS and low suction group	56.8 ± 35.6	74.2 ± 28.3	17.3 ± 7.3	0.864
High and low suction group	56.3 ± 39.2	100.2 ± 88.3	43.8 ± 49.1	0.666
Low suction only	28.4 ± 14.1	1117 ± 624.1	1089.3 ± 610	<0.001*
				
Particulate matter				
Intraoral suction group	135.1 ± 33.8	128.2 ± 22.0	−6.9 ± 11.8	0.543
EOS and intraoral suction group	136.9 ± 29.9	120.7 ± 19.7	−16.2 ± 10.2	0.456.
EOS and low suction group	128.4 ± 22.3	116.5 ± 15.9	−11.9 ± 6.4	0.573
High and low suction group	147.6 ± 30.2	132 ± 29.3	−15.6 ± 0.9	0.468
Low suction only	121.3 ± 12.6	118.2 ± 10.3	−3.1 ± 2.3	0.879

Mean ± slandered deviation through 15 min of treatment;

Statistical analysis carried out using Repeated measured ANOVA.

* P value < 0.05.

Comparing each group in the study to IOS shows evidence of statistically significant *P* value < 0.05 (Table 3A). However, there was no significant interaction between the effects of the three positions of microbial air sample devices and intervention groups in microbial airborne contamination level, *P* = 0.99.

**Table 3:** Comparison between microbial airborne contamination level (CFU/m^3^) of all intervention groups to intra-oral suction group.

**Intervention groups**	**MD (95 % CI)**	***P* value**
Intraoral suction group		
EOS and intraoral suction group	−279.4 (-480.9 – −77.9	0.007*
EOS and low suction group	−250.9 (-452.4 – −49.3	0.015*
High and low suction group	−224.9 (-426.4 – –23.3	0.029*
Low suction only	792.7 (591.2 – 994.2	< 0.001*

MD: mean difference; CI: confidence interval; IOS: Intraoral suction; EOS: extra-oral;

Statistical analysis carried out using

*P value < 0.05.

### 3.3 Particulate matter

The average and SD of PM count in the morning prior to beginning treatment for 50 days was 133.8 ± 27.2. There was no evidence of statistically significant PM among intervention groups, [*P* = 0.39]. PM reading at the end of each trial did not show any change compared to the baseline reading. The mean difference for PM in IOS was −6.9 ± 11.8, PM for EOS & IOS was −16.2 ± 10.2, PM for EOS &low suction was −11.9 ± 6.4, PM in high & low suction was −15.6 ± 0.9, PM in low suction was −3.1 ± 2.3 (Table 2 and Fig. 3B).

## 4 Discussion

In our study, EOS & IOS, and EOS & low suction showed low increase in microbial airborne contamination level compared to baseline reading. Moreover, using regular suction that including high and low suction revealed a low increase in microbial airborne contamination level related to baseline. On other hand, IOS was noticed a high increase in microbial airborne contamination level compared to baseline samples. Additionally, related to baseline reading low suction shows a very high level of microbial airborne contamination level.

All dental suctions showed different levels of increase, we compared all intervention groups to the IOS group to understand the best and worst intervention tool. Performing an additional suction device, EOS to IOS showed a great improvement in decreasing microbial airborne contamination level. While using low suction with EOS showed low microbial airborne contamination level. These findings could be indicated that using EOS was the main factor in decreasing microbial airborne contamination level.

IOS was connected to a high suction volume adaptor (DryShield, 2019), and several holes & large surface area allows water and substance to go through it although low and high suction was better in decreasing microorganism. That could be explained by the holding technique of high suction as it followed scaler movement which would result in immediate suctioning. IOS reported better findings in the decline of microorganism than low suction group. Regarding the attached IOS to high suction adaptor, the total suction volume is supposed to be higher than low suction.

Reducing CFU count during the treatment was noticed either using high suction or a HEPA filter.(Bahador et al., 2021) Both IOS and low suction shows an increase in CFU number. However, IOS reported less growth than low suction (Holloman et al., 2015), these studies' result was consistent with our findings. Other study reported the raising number of CFU through dental procedures; a higher increase was recorded when using high suction only while less CFU was observed in combining high and IOS (Suprono et al., 2021). EOS was found to be better in declining oral bacteria rather than regular oral suction (Senpuku et al., 2021). However, oral suction was not determined clearly, and the spread of oral bacteria was measured by the passive sample method and by taking swab samples from surfaces (Senpuku et al., 2021). In contrast to Desarda *et al* study, performing low suction ejector or low & high suctions were not shown any difference in CFU count (Desarda et al., 2014). These results may be related to different sampling techniques as they used a passive sample while in the present study we used an active sampling method. Additionally, the short time of the exposed plate was 20 min rather than one hour based on the standard role in the passive sampling method (Viani et al., 2020).

Possible confounding factors related to the procedure were eliminated by making a scaling procedure for all participants by one dental hygienist. Furthermore, the time of sampling and room were constant among all groups. Moreover, any participants suspected to need a specific position, additional or less time for treatment were excluded such as patients under orthodontic treatment, wearing partially removable dentures and pregnant women. The results of microbial airborne contamination level were supported by controlling all confounding factor that was related to the room which was AC microbial airborne contamination level. Additionally, confounding factors linked to participants such as age and gender were controlled. Factors related to the oral cavity which were OHI-S, DI-S and CI-S had no effect on the microbial airborne contamination level.

The present study reported no differences in PM between the intervention group. These findings could be related to the procedure that was performed as the scaling procedure did not produce solid substances such as removing restoration or grinding prosthesis procedures that possibly increase PM readings. Other studies found that high-speed handpiece produced higher particles than ultra-sonic scaler (Dudding et al., 2022, Piela et al., 2022).

A similar result was found when comparing low suction or high suction to IOS that showed indifferent results between groups, in contrast to low suction compared with high suction reported high particles regarding one sensor setup (Piela et al., 2022). These different results could be due to differences in the distance between measurement monitors and the oral cavity. Similar findings were observed in other study as low and high suction did not increase PM readings during scaling. However, high suction was better than low suction in reducing PM, the worst reading was recorded when no suction was used (Choi et al., 2022).

An additional study showed applying EOS was not change PM during the scaling procedure although employing another monitor measured PM size ranged from 0.02 −10 µm did not report any differences between EOS, low suction and high suction versus low suction and high suction except the area near to shoes cover of the dentist was higher for a group without EOS (Yang et al., 2021). In other study, the scaling procedure was completed without EOS showing high PM readings (Graetz et al., 2021). EOS when was set to 10 levels showed effective results although setting a suction on the lowest recommended level reported more effective results than non-using EOS (Chavis et al., 2021).

Different results showed in other studies, low suction was the highest group in producing particles followed by low & high suction after that EOS (Ehtezazi et al., 2021). These findings were like Matyes *et al* study, they noticed high particles with low suction followed by high suction (Matys and Grzech-Leśniak, 2020). However, both studies were done in vitro. Moreover, after adjustment of confounding factors showed that increasing in age and DI-S decreased the mean difference in PM among intervention groups. Lastly, there were few shortcoming such as observer, assistant, and participants were not blinded. Sampler's air device measuring from 0.5 µm and higher particulate size which were unable to detect smaller size. Digital dust monitor device suctioning the air volume lesser than microbial air sample device, For short period time this could not be helpful in detecting the difference regarding to reducing aerosol between oral suction devices. Moreover, the study was conducting on one dental clinic, it will be good if we can check the results in different environments (private, public, postgrad, undergrad, community based, …etc).

## 5 Conclusion

The worst oral suction device that had a negative impact on the dental environment was low suction followed by IOS. The best oral suction device was combining EOS with IOS, followed by EOS & low suction then high &low suction. Additionally, during the study, we noticed some problems that need more investigation; immediate pain in TMJ and muscles after using IOS and uncomfortable top head of EOS. Furthermore, using high suction with indirect vision was difficult for a healthcare worker who works without a dental assistant. Therefore, using EOS or IOS was more helpful for indirect vision.

## CRediT authorship contribution statement

**Abraar Bannan:** Investigation, Writing – original draft. **Iman Kamal:** Supervision, Writing – review & editing. **Naif Al Makishah:** Supervision, Writing – review & editing. **Zuhair S. Natto:** Investigation, Writing – original draft.

## Declaration of competing interest

The authors declare that they have no known competing financial interests or personal relationships that could have appeared to influence the work reported in this paper.
