# Supplementary material for: Reducing microbial airborne contamination and particulate matter using different oral suctions in dental clinic: A randomized controlled clinical trial
Source: Saudi Dent J. 2023 Nov 28;36(2):374–80. doi: 10.1016/j.sdentj.2023.11.029 (PMC10897624; doi:10.1016/j.sdentj.2023.11.029)
Supplement: Supplementary data 1 [file mmc1.pdf]

editorworld.com/admin/document/98583

Editor World
Your Words. **Perfected.**

How it works Prices Services Editors Writers FAQs Zuhair Natto

Client Console / Documents / Document

Document

Console

Documents | Messages | Submit Request | My Account | Favorites | Credit

Back | Rate Document

Title:
Banan manuscript

Original Document: EW98583.docx **Edited Document:** EW98583.docx

Client: znatto

ENG ١٠:٥٠ م ٤٥/٠٥/١١

البحث في Windows

**Editor:**	WordWorm
**Submit Date:**	10/28/23 9:06 AM
**Due Date:**	10/31/23 11:59 PM
**Service:**	3-day editing (300-30,000 words) (Editing)
**Service Type:**	Regular
**Subtotal:**	$96.99
**Discount:**	$19.40
**Total Price:**	$77.59
**Number of Words:**	3,464
**Rating:**	This document has not been rated.
**Feedback:**	

ENG ١٠:٥١ م ٤٥/٠٥/١١ البحث في Windows

**Number of Words:**	3,464
**Rating:**	This document has not been rated.
**Feedback:**	
**Coupon:**	WordWorm
**English Preference:**	American English
**Client Instructions:**	There are no comments.
**Editor Comments:**	Thank you for the assignment.

INVOICE

Editor World LLC | Your Words. Perfected.
11815 Fountain Way Ste 300
Newport News, VA 23606
Phone +1-855-511-3348
www.EditorWorld.com

INVOICE #EW98583

DATE: 10/28/2023

TO: Zuhair Natto <z_world@hotmail.com>

FOR: Editing Services

**Description**	**Amount**
Banan manuscript[3-day editing (300-30,000 words)]	$77.59
Total:	$77.59

PAID IN FULL WITH CREDIT CARD 1179

Thank you for your business!

Best regards,

The Editor World Team
